# Impact of acute stress on antimicrobial polypeptides mRNA copy number in several tissues of marine sea bass (*Dicentrarchus labrax*)

**DOI:** 10.1186/1471-2172-12-69

**Published:** 2011-12-28

**Authors:** Genciana Terova, Anna G Cattaneo, Elena Preziosa, Giovanni Bernardini, Marco Saroglia

**Affiliations:** 1 Department of Biotechnology and Molecular Sciences, University of Insubria, Via J.H. Dunant, 3 - 21100 Varese, Italy; 2 Inter-University Centre for Research in Protein Biotechnologies "The Protein Factory"- Polytechnic University of Milan and University of Insubria, Varese, Italy

## Abstract

**Background:**

In comparison to higher vertebrates, fish are thought to rely heavily on innate immune system for initial protection against pathogen invasion because their acquired immune system displays a considerably poor immunological memory, and short-lived secondary response. The endogenous antimicrobial polypeptides (AMPPs) directly and rapidly killing pathogens such as bacteria, fungi, parasites, and viruses are included within the realm of innate defenses. In addition to piscidins, AMPPs that in recent years have been shown to be commonly linked to innate defense, are histones and their polypeptide fragments, and peptides derived from the respiratory protein hemoglobin. There is evidence that a number of stresses lead to significant regulation of AMPPs and thus their monitoring could be a highly sensitive measure of health status and risk of an infectious disease outbreak, which is a major impediment to the continued success of virtually all aquaculture enterprises and is often the most significant cause of economic losses.

**Results:**

We firstly isolated and deposited in Genbank database the cDNA sequences encoding for *hemoglobin-β-like protein *(*Hb-LP*) [GeneBank: JN410659], *H2B histone-like protein 1 *(*HLP1*) GenBank: JN410660], and *HLP2 *[GenBank: JN410661]. The "*de novo*" prediction of the three-dimensional structures for each protein is presented. Phylogenetic trees were constructed on *Hb-LP*, *HLP1*, and *HLP2 *sequences of sea bass and those of other teleost, avian, reptiles, amphibian and mammalian species. We then used real time RT-PCR technology to monitor for the first time in sea bass, dynamic changes in mRNA copy number of *Hb-LP*, *HLP1*, *HLP2*, and *dicentracin *in gills, skin, eyes, stomach and proximal intestine in response to acute crowding/confinement stress. We showed that acute crowding stress induces an increase in the expression levels of the aforementioned genes, in gills and skin of sea bass, but not in other tissues, and that this expression patterns are not always rapidly reversed upon re-exposure to normal conditions.

**Conclusion:**

The higher expression of the four target genes in gills and skin of sea bass suggests that this AMPP represents a first and immediate line of defense in combating pathogens and stressors since these tissues constitute the first physiological barriers of the animal.

## Background

Antimicrobial polypeptides (AMPPs), consisting of small proteins with antimicrobial activity, are humoral components of the vertebrate and invertebrate innate immune system. In fish, the acquired immune response displays a poor immunological memory and slow lymphocyte proliferation [1,2] due to its evolutionary status [3] and poikilothermic nature. For this reason the innate immune system, which shows an instant and relatively temperature-independent response, is of primary importance in combating infections [4]. Several AMPPs have been isolated from fish, including commercially important species, such as misgurin in loach (*Misgurnus anguillicaudatus*) [5], epinecidin in grouper (*Epinephelus coioides*) [6], pleurocidin in winter flounder (*Pleuronectes americanus*) [7], moronecidin in hybrid striped bass [8], pardaxin in sole (*Pardachirus marmoratus*) [9], hepcidin in winter flounder (*Pseudopleuronectes americanus*), Atlantic salmon (*Salmo salar*), and perch (*Perca fluviatilis*) [10,11], parasin in catfish (*Parasilurus asotus*) [12], and *dicentracin *in sea bass (*Dicentrarchus labrax*) [13]. AMPPs are abundantly and typically expressed in tissues that constitute the surfaces of access for pathogen entry such as skin, gills, and the intestinal tract, [14] but they are also found in peripheral blood leukocytes and head kidney, which represents the primary organ involved in immune function [15-17].

In intensive aquaculture fish often have to cope with several conditions of chronic or severely acute stress, such as high biomass densities, that may compromise their ability to resist disease. Today, antibiotics that are effective and routinely used to treat human infections are also used for animals, either for therapy or prophylaxis [18]. However, the use of antibiotics in animal feed can promote the development of antibiotic-resistant bacteria, which can be transmitted to the general population, creating a serious worldwide crisis in both human and veterinary medicine that would urgently require new modes of antimicrobial action [14]. For this reason, the use of antibiotics in animal feed is forbidden or heavily controlled in most countries. AMPPs have potent, broad spectrum activity, directly and rapidly killing pathogens such as bacteria, fungi, parasites, and viruses. Thus, these molecules have the potential to increase resistance to infectious diseases. These diseases constitute a major constraint in aquaculture, and AMPPs contribute in a preventive manner to fish health and welfare [19].

The mechanism of action of antimicrobial peptides is strictly related to their structure: most molecules are relatively small (less than 100 aminoacid residues), and contain both hydrophilic and hydrophobic residues, enabling insertion into biological membranes [10]. Microbial killing is a consequence of the interaction of the AMPP with the microbial outer membrane, which destabilizes the membrane in a disruptive or non-disruptive manner (intracellular target) [20]. In the first case, channel formation alone promotes leakage of cytoplasmic contents, resulting in the death of the organism; in the second situation, AMPPs enter the cytoplasm, where interaction with cellular components induces the microbial killing [8].

In fish, the structure of AMPPs makes it possible for them to divide into different groups such as piscidins, histone-like proteins (HLPs), and hemoglobin-like proteins (*Hb-LP*s).

Piscidins, first isolated from mast cells of the commercially cultured hybrid striped bass (white bass, *Morone chrysops*, ♀ x striped bass, *Morone saxatilis *Walbaum ♂) [21], comprise a large number of AMPPs widely distributed in higher teleost families, such as Moronidae, Serranidae, Sciaenidae, Cichlidae, Siganidae, and Belontidae [14,22]; they are linear, amphipathic, α-helical peptides, with a highly conserved N-terminus characterized by the consensus motif I-X_5_-H-X_4_-I-H [23]. It has been demonstrated that the expression levels of piscidin 4 in gill tissue of healthy hybrid striped bass are within concentrations that are lethal to important fish pathogens [24]. Moreover, they are expressed to a significantly greater degree in healthy fish than in either nutritionally stressed or in diseased ones [25]. Gene expression of piscidins can be upregulated by integrating immunomodulators such as Bio-Mos^® ^in the food [26]; this promotes the nonspecific immune system in sea bass (*Dicentrarchus labrax*), increasing the mRNA copy number of the *dicentracin *in the head kidney [27].

Another group of broad spectrum antimicrobial molecules highly represented in fish are the histone-related AMPPs, proteins highly homologous to histones (for a review see [19]). HLPs were first isolated from the skin of channel catfish (*Ictalurus punctatus*) [28] and then identified in the skin, gills, and spleen of hybrid striped bass and rainbow trout (*Oncorhynchus mykiss*) [29,30]. HLP-1 is the most common and most potent HLP and it is highly homologous to histone H2B. Robinette and Noga [31] measured HLP-1 in channel catfish skin through an enzyme-linked immunosorbent assay (ELISA), detecting that chronic stress has a significant suppressive effect on this molecule as compared to levels in unstressed fish. The reduction in HLP-1 expression is neither affected by acute stressors nor correlated with disease status, features that -- together -- make this peptide a promising indicator for monitoring fish health. Other HLPs, histones or fragments of them, are related to the innate immune response: HLP-2, for example, which is highly homologous to histone H1, oncorhyncin II, a histone H1-derived protein [32], and parasin I, a peptide homologous to the N-terminal of human histone H2A [12], from which hipposin is also derived, isolated from skin of Atlantic halibut (*Hippoglossus hippoglossus*) [33]. The participation of histone-derived peptides in innate host defense has been demonstrated *in vivo *and *in vitro*, where HLPs and their peptide fragments are inhibitory to many important fish pathogens, including bacteria such as *Aeromonas hydrophila*, *Aeromonas salmonicida*, *Vibrio salmonicida*, *Vibrio anguillarum*, and *Vibrio alginolyticus *[19].

Like histones, whose main functions concern nuclear regulation and chromatin structure in nucleosome, the hemoglobins, which bind respiratory gasses, have also been found to be a source of peptides with potent antimicrobial activity [34]. Both intact hemoglobin tetramers and single alpha and beta subunits from various species, including human, exhibited considerable activity against gram-positive and gram-negative bacteria and fungi [35]. A variant of the β-chain of hemoglobin, Hbβ peptide 1, is expressed in skin and gill epithelium of channel catfish (*I. punctatus*) in response to the ciliate parasite ich (*Ichthyophthirius multifillis*) infection and the concentration expressed *in vivo *appeared to be well within the antiparasitic concentrations measured *in vitro *[36].

As already mentioned, reared fish often face environmental conditions that are not ideal for their physiological functions. Sometimes these conditions cause the fish to become stressed, decreasing immune capabilities and, as consequence, promote outbreaks of infections [37,38]. As a result of chronic stress AMPP expression can be downregulated [39,31] and at the same time increase disease susceptibility. In contrast, upregulation of AMPP expression can clearly protect against disease, as reported for mammals [40-42] and in a number of studies with transgenic fish [43-46]. Therefore, antimicrobial peptides could be doubly valuable in aquaculture, functioning as indicators of chronic stress to prevent disease and as a new and promising class of therapeutics.

Accordingly, in this study we investigate for the first time in *D. labrax*, the most widely reared species in Mediterranean aquaculture, the mRNA expression of different AMPPs in different tissues after acute confinement/crowding stress with the aim to relate these expression levels to the immediate early response of the innate immune system.

## Methods

### Experimental design

Forty subadult sea bass (*D. labrax*, L.) of 187.13 ± 27.5 g mean weight, obtained from Nuova Azzurro commercial hatchery (Civitavecchia, Italy) were reared, with inconsistent mortality, at a low biomass density (less than 10 kg/m^3^) in a 2.5 m^3 ^fiberglass tank connected to a water recycling system supplied with about 24 parts of water per day per day. The salinity (obtained by adding salt Oceanfish 600 LT from Prodac Int. to dechlorinated tap water) was 22 g/L.

Other water parameters were strictly controlled: temperature 21.8 ± 0.9°C, pH 7, total ammonia below 0.2 mg/L, and nitrite below 0.02 mg/L. Dissolved oxygen (DO) was maintained at over 99% of the saturation value by adding pure O_2 _to the system. A computerized multiprobe Rilheva^® ^system (Xeo4, Italy) was used to continuously monitor DO, pH, and temperature in each tank.

Fish were allowed to acclimate for 10 days before starting the experiment; then five animals (mean weight 188.8 ± 54.9 g), representing the control group, were randomly sampled. The other fish in the tank were rapidly displaced by an opaque partition which confined them to a small volume (less than 10% of the total tank volume); the biomass density was thus increased approximately tenfold (from 3 kg/m^3 ^to 29 kg/m^3^). The confinement-stress was imposed for 4 hours, after which five fish (mean weight 196.8 ± 39.6 g) were randomly sampled. The normal biomass density was then restored by removing the fiberglass partition from the tank, and other five animals were sampled after 24 hours of recovery (mean weight 175.8 ± 26.3 g). The biometric data of the animals sampled during the trial, are reported in Table 1.

**Table 1:** Mean of total length (TL), standard length (ST) and weight (W) for each group of sampled animals.

Sampled group	TL	SL	W (g)
Control	26.2 ± 2.1	21.9 ± 1.9	188.8 ± 54.9

Stressed	26.1 ± 1.3	22.2 ± 1.1	196.8 ± 39.6

Recovered	25.2 ± 1.8	22 ± 1.7	175.8 ± 26.3

For the molecular biology analysis, gills, skin, eyes, stomach, and proximal gut were isolated from fish of each group (control, stressed, and recovered) for a total of 75 samples. The tissues were immediately transferred and then stored at -80°C until the time of the analysis.

### AMPP genes isolation

#### RNA purification and first strand cDNA synthesis

Total RNA was extracted from gills, skin, eyes, stomach and proximal gut using the PureYield RNA Midiprep System (Promega, Italy) following the protocol. Briefly, 2 ml of ice-cold Lysis Solution containing β-mercaptoethanol were transferred to a 10 ml tube. Tissues of interest were excised, placed in the tube and then homogenized until no visible tissue fragments remained. Two ml of the lysate prepared above were transferred to a 15 ml centrifuge tube, and 4 ml of RNA Dilution Buffer were added. The tube was sealed, mixed thoroughly by inverting it 3-4 times, and vortex. One ml of thoroughly mixed Clearing Agent was added to the diluted lysate mixture which was then mixed inverting 2-3 times, and vortex until homogeneous. Samples were incubated at 70°C for 5 minutes to denature Tubes were then removed, and cool at room temperature for at least 5 minutes. One blue PureYield™ Clearing Column for each sample was placed in a 50 ml collection tube. Each sample was mixed by vortexing or vigorously shaking until homogeneous and the mixture was immediately poured into the assembled PureYield™ Clearing Column/collection tube. The PureYield™ Clearing Column assembly was then centrifuged in a swinging bucket rotor at 2,000 × g at 22-25°C for 10 minutes to clear the lysate. The blue Clearing Column was discarded, whereas the cleared lysate was saved in the collection tube. For additional information, please see the PureYield™ RNA Midiprep System Technical Manual #TM279, available online at: http://www.promega.com/tbs.

The quantity of the RNA was calculated at an absorbance of 260 nm. The integrity and relative quantity of RNA was assessed by electrophoresis. After extraction, 3 μg of total RNA from proximal gut was reverse transcribed into cDNA in a volume of 12 μl containing 1 μl of oligo dT16 primer (50 pmol) and 1 μl of 10 mM dNTPs. This mix was heated at 65°C for 15 min and chilled on ice, and then 4 μl of 5 × reverse transcription buffer, 2 μl of 0.1 M DTT, 1 μl RNaseOUT, and 200 units of Moloney murine Leukaemia virus reverse transcriptase were added to a final volume of 20 μl, as described in the M-MLV Reverse Transcriptase kit (Invitrogen). After incubation at 37°C for 50 min, the reaction was stopped by heating at 75°C for 15 min.

#### Cloning and sequencing

To perform PCR, an aliquot of 4 μl of the resulting cDNA was amplified with 1 μl GoTaq Polymerase (Promega) in 50 μl of final volume containing 5 μl buffer, dNTPs 10 mM, and 50 pmol of each of the designed AMPPs RT-PCR primer sets (Table 2). A total of 30 cycles (10 touchdowns) of the PCR amplification were performed for all primer sets, using an automated Thermal Cycler (Mycycler, Biorad). The annealing temperatures depended on the melting temperatures of the primer set used. An aliquot of each sample was then electrophoresed on 2% agarose gel in 1× TAE buffer (Eppendorf) and bands were detected by ethidium bromide staining. The positive control consisted in a master mix of cDNA and cytoplasmatic β-actin primers [GeneBank: AY148350] [47], while the negative control consisted of total RNA added to the RT reaction mixture without reverse transcriptase and subsequently amplified using the same set of primers and the same conditions. The negative control confirmed the absence of genomic contamination. The PCR products from AMPPs primer amplifications were cloned using the pGEM^®^-T Easy cloning vector system (Promega, Italy) and subsequently sequenced in both directions (T7 and SP6).

**Table 2:** Sequences of the primers used for molecular cloning.

Primer name	5'→3' sequence	Purpose
Hemoglobin FW1	ATGGTCCAGTGGTCAGATGC	RT-PCR
Hemoglobin RV1	CTTCAGGCTTCTTGCTGAATG	
Hemoglobin FW2	TGGACAGGGCTGTGAAGAAC	
Hemoglobin RV2	AAGTTCCTGGCTGTGGTCGT	
	
*HLP1 *FW1	AAGAAGGGCTCCAAGAAAGC	
*HLP1 *RV1	GCACTAAAGCGGTGACGAA	
*HLP1 *RV2	ACCATCACCTCCAGGGAGAT	
*HLP1 *RV3	GGCTAAACACGCAGTGTC	
*HLP1 *RV4	GGCTAAGCACGCCGTGTC	
	
*HLP2 *FW1	CCAAAGCGCCCAAGAAGA	
*HLP2 *RV1	TAAAGGCATCCTGCTGCAG	
*HLP2 *FW2	TGTCTCTCGCTGCTCTGAAG	
*HLP2 *RV2	CAAGAAGGCCAAGAAACCC	

T3 *Hb-LP sense*	*caattaaccctcactaaaggga*ATGGTCCAGTGGTCAGATGC	Standard curve
*Hb-LP antisense*	AAGTTCCTGGCTGTGGTCGT	
	
T3 *HLP1 sense*	*caattaaccctcactaaaggga*AAGAAGGGCTCCAAGAAAGC	
*HLP1 antisense*	ACCATCACCTCCAGGGAGAT	
	
T3 *HLP2 sense*	*caattaaccctcactaaaggga*CCAAAGCGCCCAAGAAGA	
*HLP2 antisense*	CAAGAAGGCCAAGAAACCC	
	
T7 *Dicentracin sense*	*gtaatacgactcactataggg*AGTGCGCCACGCTCTTTC	
*Dicentracin antisense*	CTAGTCAAAAGCTGCGCGCT	

*Hb-LP *Forward	GGTCCAGTGGTCAGATGCA	Real time RT-PCR
*Hb-LP *Reverse	ACGATCAGAAGTCTGGTCAAAGC	
*Hb-LP *Taqman^® ^probe	CCGCCATCACAAGCTGG	
	
*HLP1 *Forward	CGTGTCTAAGGTCACCAAGA	
*HLP1 *Reverse	GTACACGTAGATGGCGTAGCT	
*HLP1 *Taqman^® ^probe	CCGGCAAGAAGAAGAG	
	
*HLP2 *Forward	CCCAAGAAGAGAGCCAAGTCT	
*HLP2 *Reverse	CTGATACTGAAGGCCGCGT	
*HLP2 *Taqman^® ^probe	AAGAAGAAGACGGGTCCCTC	
	
*Dicentracin *Forward	TGCGCCACGCTCTTTCTT	
*Dicentracin *Reverse	CCCCAGGTTCAGCCATGAG	
*Dicentracin *Taqman^® ^probe	ACGACCATCGACAGCAC	

### Quantitative real-time RT-PCR

#### Generation of *in vitro*-transcribed AMPP mRNAs for standard curves

The absolute number of AMPP gene transcript copies have been quantified by comparing them with a standard graph constructed using the known copy number of mRNA of this gene. For this, a forward and a reverse primer were designed based on each mRNA sequences of the *D. labrax *genes we had identified, and on *dicentracin *cDNA sequence, already available in NCBI database [GeneBank: AY303949]. These primer pairs were used to create templates for the in vitro transcription of *Hb-LP*, *HLP1*, *HLP2 *and *dicentracin *mRNAs: the forward primers were engineered to contain a T3 phage polymerase promoter gene sequence to its 5' end and used together with the reverse primer (Table 2) in a conventional RT-PCR of total sea bass skin RNA. RT-PCR products were then evaluated on a 2.5% agarose gel stained with ethidium bromide, cloned using pGEM^®^-T cloning vector system (Promega, Italy), and subsequently sequenced in T7 and SP6 directions.

*In vitro *transcription was performed using T3 RNA polymerase and other reagents supplied in the Promega RiboProbe In Vitro Transcription System kit according to the manufacturer's protocol.

The molecular weight (MW) of the in vitro-transcribed RNA for each gene was calculated according to the following formula:

$$\text{MW}=\left({\text{n}}^{\circ }\text{ of A bases }\times \text{ 329}.\text{2}\right)\;+\;\left({\text{n}}^{\circ }\text{ of U bases }\times \text{ 30}\text{6}.\text{2}\right)\;+\;\left({\text{n}}^{\circ }\text{ of C bases }\times \text{ 30}\text{5}.\text{2}\right)\;+\;\left({\text{n}}^{\circ }\text{ of G bases }\times \text{ 345}.\text{2}\right)\;+\text{ 159}$$

The results, concentrations measured as absorbance at 260 nm and the concentration of the final working solution are reported in Table 3.

**Table 3:** Characteristics of the *in vitro*-transcribed AMPP mRNAs

mRNA	Nucleotides				MW	ng/μl	No. molecules/μl
	**A**	**U**	**G**	**C**			

***Hb-LP***	104	92	116	104	134350.2	349	1.56 × 10^12^

***HLP1***	69	44	64	71	80108.6	134	1.01 × 10^12^

***HLP2***	133	45	125	115	135969.6	271	4.68 × 10^14^

***Dicentracin***	57	53	63	66	77042.8	605.5	4.73 × 10^12^

(A: adenine; U: uracil; G: guanine; C: cytosine; MW: molecular weight). Concentration (ng/μl) was calculated by using the value of absorbance at 260 nm.

#### Generation of standard curves for AMPPs

The mRNAs produced by in vitro transcription were used as quantitative standards in the analysis of experimental samples. Defined amounts of mRNAs at 10-fold dilutions were subjected in triplicate to real-time PCR using one-step TaqMan EZ RT-PCR Core Reagents (Applied Biosystems, Italy), including 1 × Taqman buffer, 3 mM Mn(OAc)_2_, 0.3 mM dNTP except dTTP, 0.6 mM dUTP, 0.3 μM forward primer, 0.3 μM reverse primer, 0.2 μM FAM-6 (6-carboxyfluorescein-labeled probe), 5 units rTH DNA polymerase, and 0.5 units AmpErase^® ^UNG enzyme in a 25-μL reaction. AmpErase^® ^uracil-N-glycosylase (UNG) is a 26-kDa recombinant enzyme encoded by the Escherichia coli uracil-N-glycosylase gene. UNG acts on single-and double-stranded dU-containing DNA. It acts by hydrolyzing uracil-glycosidic bonds at dU-containing DNA sites. The enzyme causes the release of uracil, thereby creating an alkali-sensitive apyrimidic site in the DNA. The enzyme has no activity on RNA or dT-containing DNA. For Taqman^® ^assays, AmpErase^® ^UNG treatment can prevent the reamplification of carry over PCR products from previous PCR reactions. When dUTP replaces dTTP in PCR amplification, AmpErase UNG treatment can remove up to 200,000 copies of amplicon per 50 μL reaction. RT-PCR conditions were: 2 min at 50°C, 30 min at 60°C, and 5 min at 95°C, followed by 40 cycles consisting of 20 s at 92°C, 1 min at 62°C. The Ct values obtained by amplification were used to create standard curves for target genes.

#### AMPP transcripts quantification by one-step TaqMan^® ^real-time RT-PCR

100 ng of total RNA extracted from the experimental samples was subjected to real-time PCR, in parallel to triplicates of 10-fold-diluted defined amounts of standard mRNA, under the same experimental conditions as used to establish the standard curves. Real-time Assays-by-Design ^SM ^PCR primers and gene-specific fluorogenic probes were designed by Applied Biosystems. Primer sequences and Taqman^® ^probe of the obtained target genes are shown in Table 2.

TaqMan^® ^PCR was performed on a StepOne Real Time PCR System (Applied Biosystems). To reduce pipetting errors, master mixes were prepared to set up triplicate reactions (2 × 30 μL) for each sample.

Data from the Taqman^® ^PCR runs were collected with StepOne™ Software v2.0. CT (cycle threshold) values corresponded to the number of cycles at which the fluorescence emission monitored in real time exceeded the threshold limit. The Ct values were used to create standard curves to serve as a basis for calculating the absolute amounts of mRNA in total RNA.

#### Statistical analysis

The data were statistically compared using one-way analysis of variance (ANOVA). The level of statistical significance was set at P < 0.05.

#### *In silico *analysis

The amino acid sequences of sea bass *Hb-LP*, *HLP1*, and *HLP2 *were analyzed using the open reading frame (ORF) finder program which is available at NCBI http://www.ncbi.nlm.nih.gov. Nucleotide sequences were compared with other sequences available at the GenBank database using the BLAST algorithm [48]. Sequences were aligned using ClustalW program http://www.ebi.ac.uk/clustalw, and Multiple Sequence Alignments Editor & Shading Utility, GeneDoc, version 2.6.002 http://www.psc.edu/biomed/genedoc.

#### Protein annotation

The analysis of the primary sequences of the three target AMPPs for annotation was performed at the MemPype server http://mu2py.biocomp.unibo.it/mempype[49]. Other functional conserved domains were searched at the PROSITE http://prosite.expasy.org/[50,51] and at the Interpro 33.0 [52].

#### Tertiary structure

Because of the lack of a template for the proteins in the Protein Data Bank (PDB), http://www.pdb.org/pdb/home/home.do, a "de novo" prediction of the tertiary structures of the putative proteins was obtained at the I-Tasser server http://zhanglab.ccmb.med.umich.edu/I-TASSER. The four stages of the method implement a threading procedure, followed by structural assembly, refinement of the model and structural-based functional annotation. The output consists of five models. The first model which is the most accurate is presented in results section. The indexes of accuracy are the C-score, the TM-score and the RMSD [53-55].

We used the UCSF Chimera software, release 1.5.3 (supported by NIH P41 RR001081, http://www.cgl.ucsf.edu/chimera/) [56] to visualize, analyse and compare the structural models.

### Sites for posttranslational modifications

#### A) Phosphorylation and sumoylation

The serine, threonine and tyrosine phosphorylation sites were searched at the NetPhos 2.0 server http://www.cbs.dtu.dk/services/NetPhos/[57]; sites for sumoylation were checked with the SUMOsp 2.0 program http://sumosp.biocuckoo.org/[58].

#### B) Glycosylation

The glycosylation sites possibly present in the proteins were searched at the server of the CBS (Center for Biological Sequence Analysis, at the Technical University of Denmark, http://www.cbs.dtu.dk/services/.

Sites for the mucin-type mannosyl-O-glycosylation (Net-O-glyc 3.1) [59], C-glycosylation [60], and the epsilon-glycosylation of lysine residues [61] were searched. Sites for N-glycosylation were predicted at the Center for Biological Sequence Analysis of the University of Denmark, the CBS prediction server, http://www.cbs.dtu.dk/services/: the application automatically searches the presence of signal peptide (Net-N-glyc 1.0) [57,62]. After N-glycosylation sites were predicted, further analysis was done at the Glycosciences server http://www.glycosciences.de/modeling/. The GlyPro tool, implemented by the server, evaluates, in the .pdb file format, the availability of N-glycosylation sites not only on the basis of their consensus sequence, but also according to the accessibility of the Asn residue, whose spatial coordinates (torsion angles) are given.

#### C) DNA binding

The DNA binding sites were searched and compared in the primary structure of the two Histone-like proteins, *HLP1 *and *HLP2 *with the tools BindN+ [63] and DP- Bind [64,65].

Both are upgraded software implemented on iterative alignment across profiles of evolutionary conservation. The first tool, predicting both RNA and DNA binding, reaches prediction accuracy as high as 79% with equal sensitivity and specificity, for the DNA-binding residues. The second, dedicated to the DNA, is featured by accuracy of 77.2%, sensitivity of 76.4% and specificity of 76.6%.

### Phylogenetic analysis

The phylogenetic reconstruction was generated by the Neighbour-joining method [66], as implemented in MEGA 4.0 [67].

GenBank accession numbers for *Hb-LP *cDNA sequence comparisons are: U60902.1 [thick-tailed bushbaby (*Otolemus crassicaudatus*)]; NG000007.3 [Human (*Homo sapiens*)]; JO3642.1 [North American opossum (*Didelphis virginiana*)]; X64179.1 [Golden hamster (*Mesocricetus auratus*)]; U63145.1 [red-footed tortoise (*Geochelone carbonaria*); M73995.1 [Red junglefowl (*Gallus gallus*)]; GQ272085.1 [Speckled teal (*Anas flavirostris flavirostris*); X03142.1 [African clawed frog (*Xenopus laevis*)]; EU877979.1 [Marine toad (*Bufo marinus*); JN410659 [Sea bass (*Dicentrarchus labrax*)]; AB043642.1 [Japanese amberjack (*Seriola quinqueradiata*)]; AY190698.1 [Red seabream (*Pagrus major*)]; AJ277207.1 [Seabream (*Sparus aurata*)]; DQ109665.1 [Red drum (*Sciaenops ocellatus*)].

GenBank accession numbers for *HLP1 *cDNA sequence comparisons are: XM844635.2 [Dog H2B (*Canis lupus familiaris*)]; XM002930226.1 [Giant panda (*Ailuropoda melanoleuca*)]; XM_001505029.2 [Horse (*Equus caballus*)]; XM_001367484.1 [Gray short-tailed opossum (*Monodelphis domestica*)]; XM_001515033.1 [Platypus (*Ornithorhynchus anatinus*); XM_003259153.1 [Northern white-cheeked gibbon (*Nomascus leucogenys*)]; NM_080593.2 [Human (*Homo sapiens*)]; XM_003311114.1 [Chimpanzee (*Pan troglodytes*); XM_003416418.1 [African savanna elephant (*Loxodonta africana*)]; AB124798.1 [Japanese gliding frog (*Rhacophorus schlegelii*)]; XM_003226650.1 [Green anole (*Anolis carolinensis*)]; XM_427116.1 [Red jungle fowl (*Gallus gallus*)]; XM_003201926.1 [Turkey (*Meleagris gallopavo*)]; JN410660 [Sea bass (*Dicentrarchus labrax*)]; GU982558.1 [orange-spotted grouper (*Epinephelus coioides*)]; X02916.1 [Rainbow trout (*Onchorhynchus mykiss*)]; AB435638.1 [Japanese medaka (*Oryzias latipes*)]; XM_002666939.1 [Zebrafish (*Danio rerio*)].

GenBank accession numbers for *HLP2 *cDNA sequence comparisons are: XM_002194486.1 [zebra finch (*Taeniopygia guttata*)]; M17018.1 [red jungle fowl (*Gallus gallus*)]; X06128.1 [duck (*Anas platyrhynchos*)]; NM_001016946.2 [western clawed frog (*Xenopus (Silurana) tropicalis*)]; AY158905.1 [mouse (*Mus musculus*)]; XM_002721618.1 [rabbit (*Oryctolagus cuniculus*)]; XM_001364664.1 [gray short-tailed opossum (*Monodelphis domestica*)]; XM_545380.1 [dog (*Canis lupus familiaris*)]; XM_527252.2 [chimpanzee (*Pan troglodytes*); NM_005325.3 [human (*Homo sapiens*)]; JN410661 [sea bass (*Dicentrarchus labrax*)]; AY184811.1 [goldfish (*Carassius auratus*)]; XM_693405.3 [zebrafish (*Danio rerio*)]; X02624 [rainbow trout (*Salmo gairdneri*) (*Onchorhynchus mykiss*)].

## Results

### Sea bass AMPPs cDNA sequencing

At the beginning of this research, the cDNA sequence coding for sea bass *dicentracin *was available at the NCBI Genbank database [GeneBank: AY303949] [13]. The coding sequences for the other three target genes: Hemoglobin-β like protein (*Hb-LP*), H2B histone like protein 1 (*HLP1*) and H1 histone like protein 2 (*HLP2*), conversely, were not available in public databases. Thus, a BlastN search was performed http://www.ncbi.nlm.nih.gov/BLAST/ on the complete, nonredundant Genbank nucleotide database for orthologues of these genes in other fish species. A multiple sequence nucleotide alignment http://www.ebi.ac.uk/clustalw was then carried out on coding sequences and a strategy based on regions of strong nucleotide conservation was used to design the primers.

In the case of *Hb-LP*, the coding sequence was amplified using two sets of primer designed on conserved regions within the coding sequences of sea bream (*Sparus aurata*) [GeneBank: AJ277207] and rainbow trout (*O. mykiss*) [GeneBank: NM_001124187]. With these primer pairs (Hemoglobin forward1 + Hemoglobin reverse1 and Hemoglobin fw2 + Hemoglobin rv2), (Table 2) we obtained two cDNA fragments, of 201 and 416 base pairs (bp), respectively. The 416 bp fragment was then deposited in GenBank under the [GeneBank: JN410659]. The deduced amino acid sequences which is encoded by this fragment, is composed of 138 amino acids, of a calculated molecular mass of approximately 15 kDa.

For *HLP1*, we designed different primer pairs based on the alignment of the sequences of *Danio rerio *[GeneBank: BC163428], *Osmerus mordax *[GeneBank: BT075469] and *Oryzias latipes *[GeneBank: AB435638]. Among the primer pairs which we tested, *HLP1 *Fw1 + HPL1 RV2 (Table 2) gave a cDNA fragment of 248 bp, corresponding to the target gene. This partial coding sequence was deposited in GenBank under the accession number JN410660. Deduced amino acid sequence encoded by sea bass *HLP1 *is composed of 82 amino acids, of a calculated molecular mass of approximately 9 kDa.

*HLP2 *coding sequence was obtained with the same strategy of *Hb-LP*, with primer designed on conserved regions within the sequences of *Salmo gairdnerii *[GeneBank: X02624], *Carassius auratus *[GeneBank: AY184811] and *D. rerio *[GeneBank: BC183478]. The primers pair HPL2 FW1 + HPL2 RV2 amplified a cDNA fragment of 412 bp representing the partial coding sequence, which was deposited in GenBank under the accession number JN410661. Deduced amino acid sequence is composed of 138 amino acid, and has a calculated molecular mass of approximately 14 kDa.

### Creation of standard curves for absolute quantitation of *Hb-LP*, *HLP1*, *HLP2 *and *dicentracin*

The correct template lengths, including the T3 promoter, were verified by 2.5% agarose gel electrophoresis. Quality and purity of mRNAs were confirmed by the ratio of absorptions at 260/280 nm, i.e., 1.8-2.0.

To obtain threshold cycle (Ct) values for the target genes, defined quantities at 10-fold dilutions of *in vitro *transcribed mRNAs were subjected to a one-tube two-step real-time RT-PCR. The standard curve created for *Hb-LP*, *HLP1*, *HLP2 *and *dicentracin *was based on the linear relationship between the Ct value and the logarithm of the starting amount.

### AMPP expression patterns in response to acute stress

**Figure 1:**
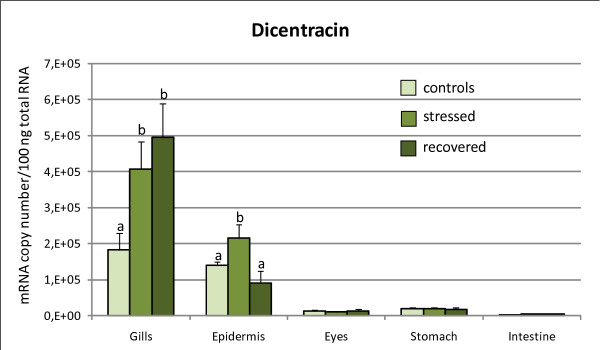
***Dicentracin *gene expression levels in tissues of *D. labrax***. The *dicentracin *mRNA copy number was measured by real-time PCR in gills, skin, eyes, stomach and gut, and it was normalized as a ratio to 100 ng total RNA. Fish were sampled before stress (control), after 4 hours of confinement/crowding (stress), and then after 24 hours of recovery from stress. The means of five animals in each group are shown. Bars indicate standard error of the mean. Differences were determined by one-way ANOVA. Different letters indicate significantly different means (p < 0.05) between control, stressed and recovered fish groups.

**Figure 2:**
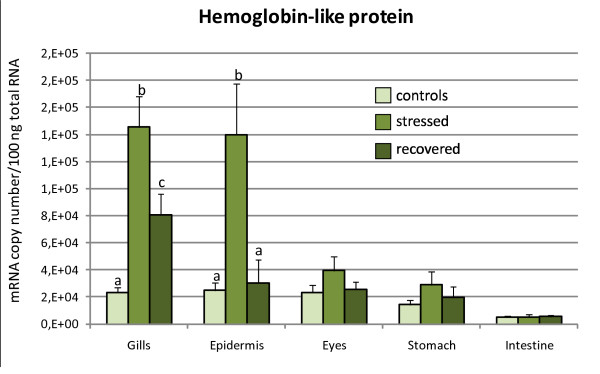
***Hb-LP *gene expression levels in tissues of *D. labrax***. The *Hb-LP *mRNA copy number was measured by real-time PCR in gills, skin, eyes, stomach and gut, and it was normalized as a ratio to 100 ng total RNA. Fish were sampled before stress (control), after 4 hours of confinement/crowding (stress), and then after 24 hours of recovery from stress. The means of five animals in each group are shown. Bars indicate standard error of the mean. Differences were determined by one-way ANOVA. Different letters indicate significantly different means (p < 0.05) between control, stressed and recovered fish groups.

**Figure 3:**
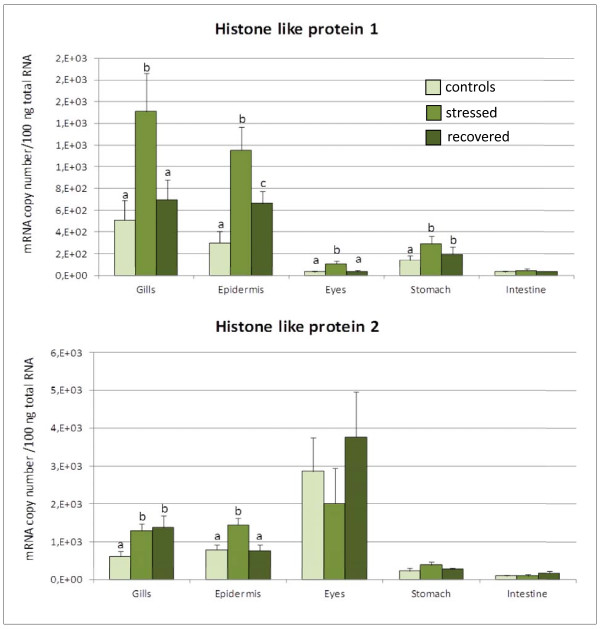
***HLP1 and HLP2 *gene expression levels in tissues of *D. labrax***. The *HLP1 and HLP2 *mRNA copy number was measured by real-time PCR in gills, skin, eyes, stomach and gut, and it was normalized as a ratio to 100 ng total RNA. Fish were sampled before stress (control), after 4 hours of confinement/crowding (stress), and then after 24 hours of recovery from stress. The means of five animals in each group are shown. Bars indicate standard error of the mean. Differences were determined by one-way ANOVA. Different letters indicate significantly different means (p < 0.05) between control, stressed and recovered fish groups.

Total RNA from sea bass tissues was subjected to real-time RT-PCR using the standard curve method to determine absolute amounts of each target gene mRNAs in these tissues. This analysis revealed that the AMPPs being considered are mostly expressed in gills and skin (Figures 1, 2, 3), where we also observed a significant change in their expression after acute stress. After four hours of confinement stress at high biomass density the mRNA levels of *dicentracin*, *Hb-LP*, and *HLP2 *significantly increased in gills and skin as well, but we did not detect any variation in their expression in stomach, eyes, and gut in any of the tested groups (Figures 1, 2, 3). On the contrary, *HLP1 *expression was upregulated after confinement stress in all the tissues examined, except for the gut (Figure 3).

After 24 hours of recovery, the levels of *dicentracin *and *HLP2 *mRNA in the gills were still high, whereas in the skin the expression had returned to the control group values (Figure 1). *Hb-LP *mRNA levels decreased in gills and skin of the "recovered" group, reaching the control value only in the skin (Figure 2). *HLP1 *gene expression decreased in gills, skin, stomach, and eyes as compared to the expression levels of the stressed group, but only in the gills and eyes did it return to the control group levels (Figure 3).

#### *In silico *analysis

A complete set of bioinformatics analysis was carried out to characterize the three AMPPs isolated in *D. labrax *and here described.

### Protein annotation

The AMPPs here considered are deprived of signal peptide, transmembrane domains and glycosylphosphatidylinisotol (GPI)-anchor. Annotation at the Mempype server classifies them as globular protein; the *Hb-LP *is strongly predicted to be localized on internal membrane, and the *HLP1 *and 2 in the chromosomes or nucleus.

According to the Gene Ontology (GO) entries, the *Hb-LP *belongs to the hemoglobin complexes, devoted to oxygen transporter activity in response to hypoxia.

The GO predicts for the *HLP2 *a very high probability of binding (near 100%) for the DNA, and weaker (70% ca.) for the proteins. Among the other biological processes mediated by this protein, the spermatogenesis is the most probable (50%).

The second histone-like, *HLP1*, is instead better associated with protein binding (70% ca) than with DNA (no more than 20-30%). Its role in cellular response seems to be better defined in the field of defense response to bacterial (either Gram-negative or Gram-positive) infections mediated by the migration of mononuclear cell migration and by the activation of the plasminogen (50% ca.).

The amino acid sequence of the *Hb-LP *contains a conserved motif, which is localised between the 5^th ^and 138^th ^amino acids. This motif aligned with a score of 32.953 with the globin classified at the PROSITE entry as PS01033. Two metal binding residues are also present at positions H64 and H93.

The linker histone H1/H5 motif (hit PS1504 in PROSITE, score 23.208) is present in the chain of the *HLP2*, between the amino acids 14^th ^and 87^th^, whereas the *HLP1 *does not contain any conserved motifs.

### Tertiary structure

**Figure 4:**
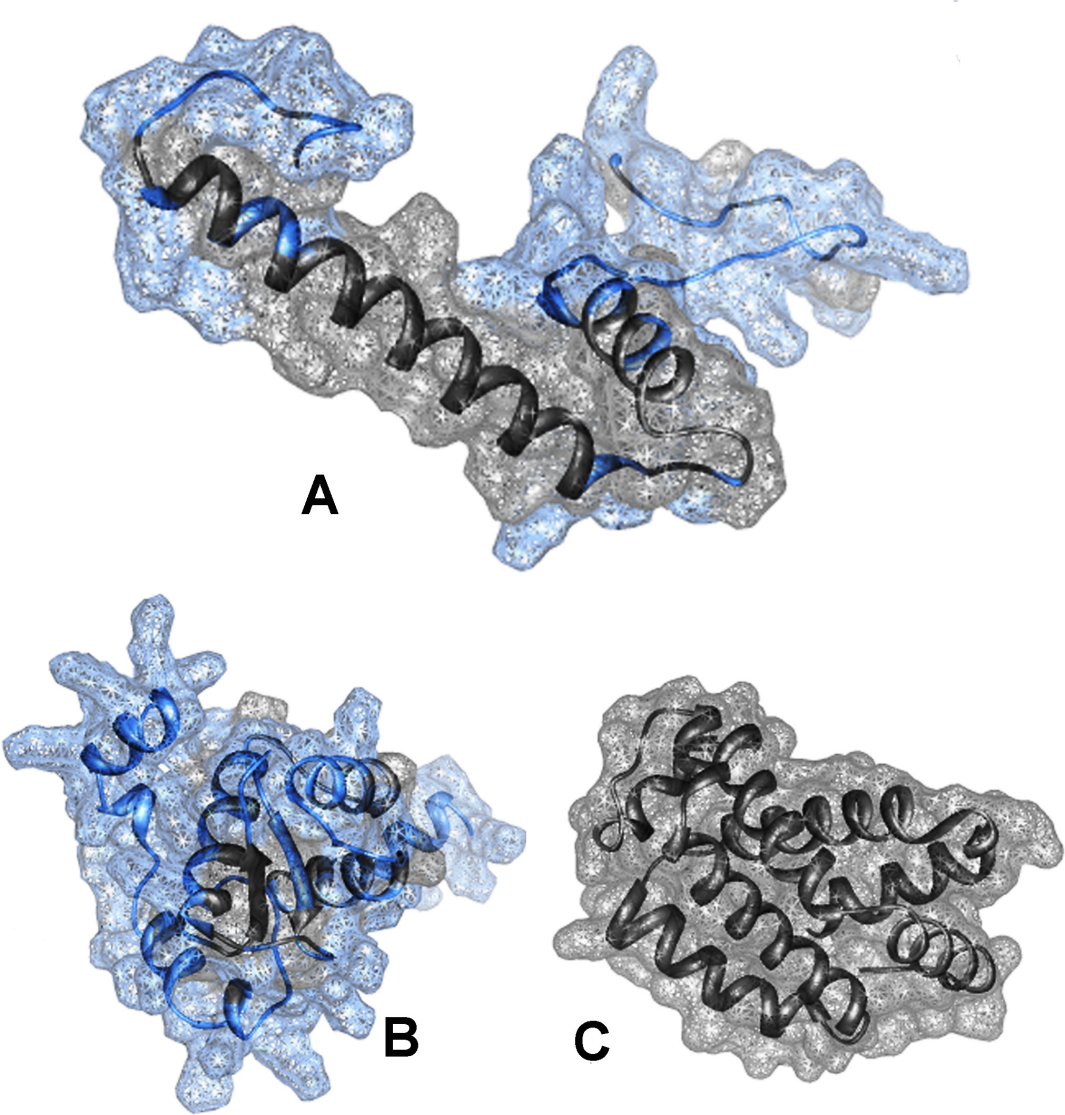
**DNA binding sites of the "de novo" predicted tertiary structure of *D. labrax *AMPPs**. (*HLP1 *is A, *HLP2 *is B and HB-like protein is C). DNA binding sites are presented in light blue with the surface.

According to their globular nature, the predicted tertiary structure of the AMPPs of *D.labrax *is dominated by ordered alpha helices (Figure 4); two antiparallel beta strands are present in the HPL2 (Figure 4B). I-Tasser server predicted these structure with different degree of accuracy, the C-score was high for Hb-like (C-score: 1.35, TM-score: 0.90 ± 0.06; RMSD: 2.0 ± 1.6 Å), and *HLP1 *(C-score: 0.05; TM-score: 0.72 ± 0.11; RMSD: 3.5 ± 2.4 Å), and lower for *HLP2 *(C-score: -2.57; TM-score: 0.42 ± 0.14; RMSD: 10.3 ± 4.6).

### Sites for posttranslational modifications

**Figure 5:**
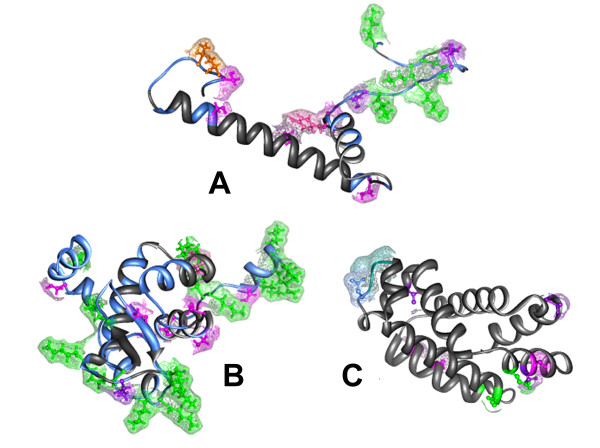
**The functional sites of the "de novo" predicted tertiary structure of D. labrax AMPPs**. (*HLP1 *is A, *HLP2 *is B and HB-like protein is C). Colour code: residues predicted to be phosphorylated: pink = Y, magenta = S, purple = T; epsilon-glycosylated = green; N-glycosylated = light blue (only in the *Hb-LP*); bound to DNA = blue (only in *HLP1 *and *HLP2*). The Thr residues shown in orange in *HLP1 *can be phosphorylated or O-glycosylated.

No sumoylation sites are present on the three AMPPs, but they could eventually undergo phosphorylation and glycosylation: the predicted sites are shown in Figure 5.

Among the different types of glycosylation, the C-mannosylation was never predicted, whereas the O-GalNAc (mucin type) glycosylation sites were found only in HPL1. They involve two threonine residues (T77 and T79) near the C-terminus which could eventually undergo phosphorylation. The prediction scores for T77 are fairly over the threshold for both the processes, whereas the phosphorylation score for T79 is higher (0.976 vs 0.545, threshold: 0.5).

A motif allowing the N-glycosylation is present only in the *Hb-LP*, and it is centred on the residue N48 (_48_NLS_50_). Table 4 reports the spatial coordinates of this site on the tertiary structure of the protein, as obtained at the Glycosciences server http://www.glycosciences.de/modeling/.

**Table 4:** Coordinates of N-glycosylation site.

PDB residue	Torsion angle (0°- 360°)			
	
	N_CA_CB_CG	CA_CB_CG_OD1	C1_ND2_CG_CB	O5_C1_ND2_CG
48 N	300°	340°	160°	260°

This site was predicted at residue 48 N of *Hb-LP *by the GlyPro at the Glycoscience server.

The most common type of glycosylation among those predicted at the CBS server occurring on the three AMPPs is the epsilon-type of lysine residues (Figure 5). The process seems to extensively involve the *HLP2 *chain (Figure 5B) and the N-terminus of the *HLP1 *(Figure 5A) with generally high scores (range: 0.906-0.953 in the *Hb-LP*, 0.523-0.940 in the *HLP2 *and 0.721-0.909 in *HLP1*).

### Phylogenetic analysis

**Figure 6:**
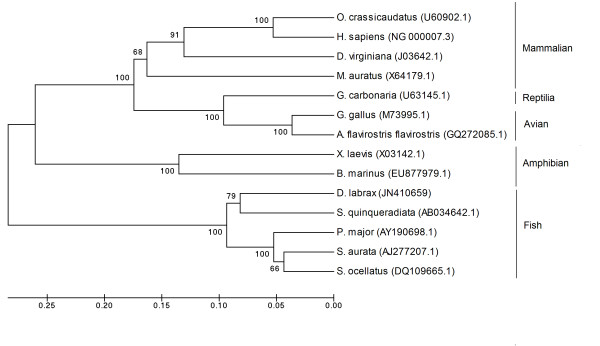
**Phylogenetic tree comparing the sequence of *Hb-LP *in sea bass (*Dicentrarchus labrax*) with those of other vertebrate species**. The scale bar refers to evolutionary distances in substitutions per site. The numbers at tree nodes refer to percentage bootstrap values after 1000 replicates. GenBank accession numbers for *Hb-LP *cDNA sequence comparisons are: U60902.1 [thick-tailed bushbaby (*Otolemus crassicaudatus*)]; NG000007.3 [human (*Homo sapiens*)]; JO3642.1 [North American opossum (*Didelphis virginiana*)]; X64179.1 [golden hamster (*Mesocricetus auratus*)]; U63145.1 [red-footed tortoise (*Geochelone carbonaria*); M73995.1 [red junglefowl (*Gallus gallus*)]; GQ272085.1 [speckled teal (*Anas flavirostris flavirostris*); X03142.1 [african clawed frog (*Xenopus laevis*)]; EU877979.1 [marine toad (*Bufo marinus*); JN410659 [sea bass (*Dicentrarchus labrax*)]; AB043642.1 [Japanese amberjack (*Seriola quinqueradiata*)]; AY190698.1 [red seabream (*Pagrus major*)]; AJ277207.1 [seabream (*Sparus aurata*)]; DQ109665.1 [red drum (*Sciaenops ocellatus*)].

To analyse the evolutionary relationship of sea bass *Hb-LP*, *HLP1*, and *HLP2 *with respect to other publicly available, related genes in teleost, avian, amphibians, reptiles, and mammalian species, we reconstructed a phylogenetic tree for each gene (Figure 6).

The clustering pattern provides evidence that sea bass Hb-like (Figure 6) is grouped with high bootstrap support in the lineage of other teleosts, sharing the highest similarity with Japanese amberjack *Hb-LP *(82%), sea bream (80%), red drum (80%), and red sea bream (78%) whereas the avians (red jungle fowl, speckled teal), amphibians (african clawed frog, marine toad), reptiles (red-footed tortoise) and mammalians (thick-tailed bushbaby, human, opossum, golden hamster) are grouped into other distinct lineages.

**Figure 7:**
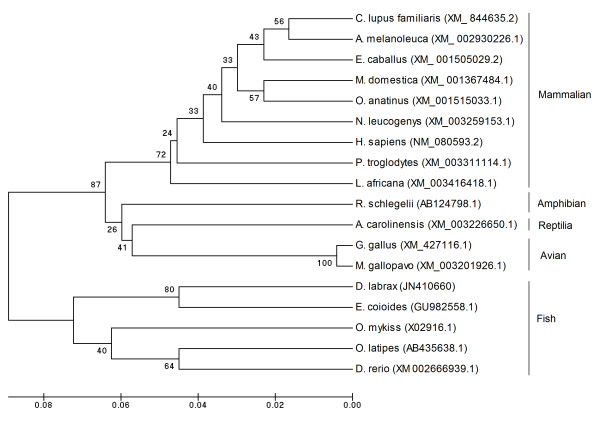
**UPGMA phylogenetic tree depicting the evolutionary relationship of vertebrate *HLP1 *proteins**. The tree was constructed using the neighbour-joining method based on the alignment of the complete cDNA coding sequences of known vertebrate *Hb-LP*s. The scale bar refers to evolutionary distances in substitutions per site. Bootstrap values (1000 replicates) indicating the occurrence of nodes are reported above each branch in the figure. GenBank accession numbers for *HLP1 *cDNA sequence comparisons are: XM844635.2 [Dog H2B (*Canis lupus familiaris*)]; XM002930226.1 [Giant panda (*Ailuropoda melanoleuca*)]; XM_001505029.2 [Horse (*Equus caballus*)]; XM_001367484.1 [Gray short-tailed opossum (*Monodelphis domestica*)]; XM_001515033.1 [Platypus (*Ornithorhynchus anatinus*); XM_003259153.1 [Northern white-cheeked gibbon (*Nomascus leucogenys*)]; NM_080593.2 [Human (*Homo sapiens*)]; XM_003311114.1 [Chimpanzee (*Pan troglodytes*); XM_003416418.1 [African savanna elephant (*Loxodonta africana*)]; AB124798.1 [Japanese gliding frog (*Rhacophorus schlegelii*)]; XM_003226650.1 [Green anole (*Anolis carolinensis*)]; XM_427116.1 [Red jungle fowl (*Gallus gallus*)]; XM_003201926.1 [Turkey (*Meleagris gallopavo*)]; JN410660 [Sea bass (*Dicentrarchus labrax*)]; GU982558.1 [orange-spotted grouper (*Epinephelus coioides*)]; X02916.1 [Rainbow trout (*Onchorhynchus mykiss*)]; AB435638.1 [Japanese medaka (*Oryzias latipes*)]; XM_002666939.1 [Zebrafish (*Danio rerio*)].

Sea bass *HLP1 *(Figure 7) is also grouped with high bootstrap support in the lineage of other teleosts, sharing the highest similarity with orange-spotted grouper *HLP1 *(86%), Japanese medaka (86%), rainbow trout (82%), and zebrafish (81%). Sea bass *HLP1 *shares a similarity of 82% and 83% with avians such as red jungle fowl and turkey, respectively; of 84% with amphibians (Japanese gliding frog); of 81% with reptiles (green anole), and of 77-81% with mammalians (dog, giant panda, horse, gray short-tailed opossum, platypus, northern white-cheeked gibbon, human, chimpanzee, and African savanna elephant).

**Figure 8:**
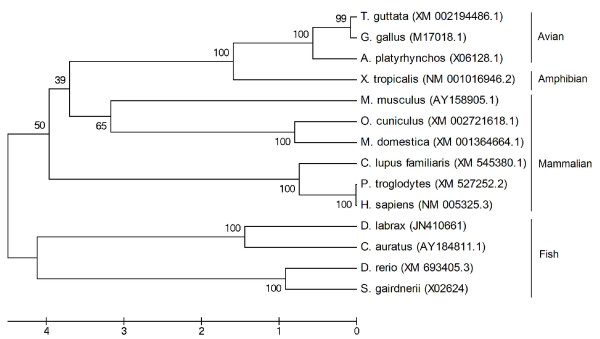
**Phylogenetic tree comparing the sequence of *HLP2 *in sea bass (*Dicentrarchus labrax*) with those of other vertebrate species**. The scale bar refers to evolutionary distances in substitutions per site. The numbers at tree nodes refer to percentage bootstrap values after 1000 replicates. GenBank accession numbers for *HLP2 *cDNA sequence comparisons are: XM_002194486.1 [zebra finch (*Taeniopygia guttata*)]; M17018.1 [red jungle fowl (*Gallus gallus*)]; X06128.1 [duck (*Anas platyrhynchos*)]; NM_001016946.2 [western clawed frog (*Xenopus (Silurana) tropicalis*)]; AY158905.1 [mouse (*Mus musculus*)]; XM_002721618.1 [rabbit (*Oryctolagus cuniculus*)]; XM_001364664.1 [gray short-tailed opossum (*Monodelphis domestica*)]; XM_545380.1 [dog (*Canis lupus familiaris*)]; XM_527252.2 [chimpanzee (*Pan troglodytes*); NM_005325.3 [human (*Homo sapiens*)]; JN410661 [sea bass (*Dicentrarchus labrax*)]; AY184811.1 [goldfish (*Carassius auratus*)]; XM_693405.3 [zebrafish (*Danio rerio*)]; X02624 [rainbow trout (*Salmo gairdneri*) (*Onchorhynchus mykiss*)].

Phylogenetic analysis at cDNA levels supported the evolutionary conservation of *HLP2 *(Figure 8) in fish species (*D. labrax*, *C. auratus*, S. *gardneri *(*O.mykiss*), and *D. rerio*). Sea bass *HLP2 *shares a similarity of 56% with godfish, rainbow trout and zebrafish *HLP2*; of 52% with avians (zebra finch, red jungle fowl, and duck), of 53% with amphibians (Western clawed frog), and a similarity of 50-55% with the mammalians (mouse, rabbit, gray short-tailed opossum, dog, chimpanzee, and human).

## Discussion

To be profitable, aquaculture must optimize rearing conditions to grow fish in as little space and at as fast a rate as possible; however, this high-density rearing condition creates a stressful environment not suitable for several physiological functions of fish. Thus, preventive strategies to control the health and immune responses of the fish play a crucial role in aquaculture.

AMPPs can be used as sensitive indicators of fish health [19]. They are mainly present at sites of potential entry of pathogens and their levels vary significantly even before an animal shows signs of disease, making it possible to intervene before adverse conditions cause overt disease and subsequent loss.

*HLP1 *and 2 are the most known AMPPs in fish and from the literature we could deduce that, given their ubiquity, they can carry out functions related to defense mechanisms in most teleosts [19], including *D. labrax*. Regarding *Hb-LP*s, it has been shown that their cytosolic breaking can generate peptides that possess biological activity related to hypothetical, secondary functions in different tissues, such as temperature regulation, antibacterial and inflammatory activity, blood pressure regulation, and enhancement of cholinergic transmission [68]. *Dicentracin*, recently isolated in *D. labrax *[13], has a role in immune defense. Using *in situ *hybridization assay, Salerno et al. [13] observed *dicentracin *expression in 68-71% of peripheral blood leukocytes, kidney leukocytes or peritoneal cavity leukocytes, whereas *dicentracin *mRNA was observed in most of the granulocytes, as well as in monocytes from both peripheral blood and head kidney, and in macrophages from peritoneal cavity.

The predicted 3D structure of the AMPPs in our study is dominated by ordered alpha helices. Most AMPP molecules are frequently alpha-helical, with an overall positive charge (generally +2 to +9) imparted by the presence of multiple lysine and arginine residues and a substantial portion (30% or more) of surface hydrophobic residues. These features seem to play an essential role in the various mechanisms of membrane permeabilization, which often lead to microbial cell death [69]. Microbial killing is a consequence of the interaction of the AMP with the microbial outer membrane, which destabilizes the membrane and promotes channel formation. A first step in this mechanism of action is the electrostatic interaction between the cationic peptide and the negatively charged components of the membrane of the pathogen; hence, an increase in positive charge of the peptide will increase microbicidal activity [70].

However, membrane permeabilization, *per se*, does not invariably result in bacterial death. Although AMPs interact with microbial cell membranes, creating pores that lead both to leakage of ions and metabolites, and depolarization, there is increasing evidence to indicate that these effects are not the only mechanisms of microbial killing, and that antimicrobial peptides have other intracellular targets [20]. Some peptides must cross the cytoplasmic membrane and they have developed unique mechanisms to translocate to the cytoplasm. Once in the cytoplasm, translocated peptides can inhibit nucleic-acid synthesis, alter the cytoplasmic membrane septum formation, inhibit cell-wall synthesis, inhibit protein synthesis or inhibit enzymatic activity. An example is buforin II, a histone H2A-derived antimicrobial peptide, which inhibits the cellular functions of *E. coli *by binding to DNA and RNA after penetrating the cell membranes.

PR-39, a proline/arginine-rich antimicrobial peptide, is able to bind the cell membrane of *E. coli *without affecting its integrity and kill bacteria by blocking both DNA and protein synthesis. Tachyplesin binds in the DNA minor groove. At their minimal inhibitory concentrations, pleurocidin and dermaseptin both inhibit nucleic acid and protein synthesis without damaging the *E. coli *cytoplasmic membrane. *E. coli *cells exposed to these peptides are unable to undergo cell division due to the blocking of DNA replication or the inhibition of membrane proteins that are involved in septum formation [20,71].

In fish, the tertiary structure of at least two AMPPs of the group of "piscidins" has been resolved by crystallography: [piscidin 1: Protein Data Bank entry 2O JM, and hepcidin: PDB entry 3 HOT http://www.pdb.org/pdb/home/home.do]. Piscidin 1, isolated from hybrid striped bass (*Morone saxatilis *x *M. chrysops*), is very similar to dicentracin from sea bass (*Dicentrarchus labrax*). According to Campagna et al., [72] which determined the three-dimensional structure of piscidin, the number of positively charged residues (two arginines, one lysine, and four histidines), and the ability to form an amphipathic helical structure in membrane mimicking environments, are the two main features responsible for the antimicrobial activity of this AMPP. Furthermore, the substitution of two glycine residues, Gly^8 ^and Gly^13^, with Ala or Pro on piscidin's structure decrease the bacterial cell selectivity of this antimicrobial peptide [73]. In particular, the antimicrobial and haemolytic activities, and the ability to permeabilize the model phospholipid membranes, were higher in piscidin with Gly or Pro at position 8 than for its counterparts with either Gly or Pro at position 13 [73].

The other peptide of the "piscidin" group, hepcidin, reveals a distorted beta-sheet shape with a hairpin loop. The beta-sheet structure is stabilized by disulphide pairing of Cys residues and hydrogen bonding between the two antiparallel strands [74]. This leads to a markedly amphipathic peptide structure, a hallmark of many antimicrobial and antifungal peptides. The hepcidin 3D conformation is important for its aggregation and function. In particular the proximity of the loop portion of the peptide to the rest of the peptide is a feature associated with the aggregation of the peptide, whereas the rare vicinal disulphide pairing in the hairpin loop may be a significant characteristic in the function of this peptide [74].

The tertiary structure of other groups of AMPPs such as "Hemoglobin like" and "Histone-like" have not yet been resolved by crystallography in fish. Thus, the structures of Hb-by crystallography in fish. Thus, the structures of Hb-*LP*, *HLP1*, and *HLP2 *were "de novo" predicted in our study. These structures shows a distribution of hydrophobic residues at the surface, suggesting a functional involvement of such residues. This is similar to the aforementioned 3D structures of piscidin 1, and hepcidin. Indeed, sea bass Hb-*LP *and *HLP1 *align partially to the alpha helix of piscidin 1, whereas *HPL2 *aligns partially to the chain C of hepcidin. Furthermore, the charge distribution of the aligned fragments is very similar, suggesting similar functional properties associated to the 3D structure of these AMPPs.

Antimicrobial peptides of animal origin display different types of post-translational modifications that can modify their activity in a significant way; among the most frequent, are glycosylation, and phosphorylation. The analysis of the sea bass AMPP sequences show the presence of O-GalNAc glycosylation sites in *HPL1*, whereas a motif allowing the N-glycosylation is present in the *Hb-LP*.

Aside from its important role in antimicrobial activity, little is known about other roles of glycosylation in the lethal mechanisms of the corresponding peptides, though some ideas have been advanced, such as protection against proteinases, modification of secondary structure inhibition of enzymes involved in peptidoglycan biosynthesis or specific recognition between pathogen and peptide [70]. Phosphorylation has been described for histatins although absence of phosphate does not preclude candidacidal activity of this antimicrobial peptide. Other AMPPs such as chromacin, requires both O-glycosylation and tyrosine phosphorylation for full antibiotic activity, and the synthetic nonmodified peptide is completely inactive. Enkelytin, an antibacterial peptide derived from proenkephalin A has two phosphoserines and an oxidized methionine required for activity [70].

After isolating the sea bass cDNAs of *HLP1*, *HLP2*, and *Hb-LP*, we quantified the expression of these genes and of that of *dicentracin *(previously isolated by Salerno et al. [13]) by using real-time RT-PCR in the skin, gills, eyes, stomach, and proximal intestine of three groups of animals. The first group represented the control conditions; the second one was subjected to acute confinement/crowding stress; and the third one was subjected to the same acute stress followed by 24 hours of recovery. Reverse transcription-real-time PCR, which is based on two sequential reactions -- reverse transcription of the mRNA, followed by applying the resulting cDNA as a PCR template -- is considered as one of the most accurate methods for transcript evaluation [75]. In aquaculture, the use of real-time PCR has recently expanded, in particular for detecting microbes, parasites, and genetically modified organisms. However, any need for fast and precise measurements of small amounts of nucleic acids in fish represents a potential niche for real-time PCR-based applications, and as machines become faster, cheaper, smaller, and easier to use, more in-field application needs for this technology in aquaculture are likely to be filled.

The results obtained for the *Hb-LP *gene showed an increase in the number of mRNA copies in gills and skin of stressed fish as compared to control animals. During the recovery phase, the mRNA copy number of this *Hb-LP *tends to decrease in both tissues, reaching the control values after 24 hrs of recovery only in skin (Figure 2). *Hb-LP *expression observed in the gills and skin was higher than in eyes, stomach, and gut. The upregulation of the transcription could be considered as a response for coping with acute stress and it could be associated with an increase in *Hb-LP *protective activity against noxious external agents. An increase in the antibacterial activity of a hemoglobin-related AMPP, the hemoglobin-like protein 1 (HbβP-1), has been observed in gills and skin of catfish (*I. punctatus*) after exposure to ectoparasite *I. multifillis *[36], and subsequent studies [76] showed that HbβP-1 is active against another ectoparasite, *Amyloodinium ocellatum*. This microorganism is also the target of the histone-like protein isolated from the gills of rainbow trout (*O. mykiss*), catfish, and sea bass [29,30]. We could deduce that HbβP-1 and its related peptides may act in association with other AMPPs, such as histone-like proteins, isolated from the same tissues, creating a powerful line of innate defense against pathogens. No variation in transcript levels in the other analyzed tissues might indicate that this gene is constitutively expressed and performs its function in immune defense proactively against a stressful event. However, other antimicrobial molecules might play immunological roles in the eyes, stomach, and gut; in fact, a single organism can possess different classes of AMPPs and several variants of the same class [15]. The induction of transcription of *Hb-LP *gene after four hours of crowding stress, compared to the control samples, is consistent with the data of Liu et al. [77], who showed that the induction of β-globin requires 3.5 to 24 hours in the presence of interferon or lipopolysaccharide.

The results obtained for *HLP1 *and *HLP2 *confirmed that the acute crowding stress induces AMPP transcription and the recovery phase does not always restore mRNA levels to the control values (Figure 3). Thus, the histone-like proteins may also be involved in the immediate early response of the innate immune system. Both *HLP1 *and *HLP2 *were expressed mainly in the gills and skin, where a statistically significant change in the number of mRNA copies was observed between control and stressed animals. *HLP2 *expression was also relevant in eyes, organs that may be damaged by wounds and traumas under conditions of high livestock density. The expression of HLPs in the other tissues, such as gut, could be constitutive, as mRNA levels did not change following acute stress; however, the antimicrobial action in those tissues might be performed by other AMPPs that work together with those analyzed in this work.

Richards et al. [78] reported that *HLP2 *isolated from the gut, stomach, and liver of Atlantic salmon (*S. salar*) was involved in specific immune responses. The authors showed that this protein has antimicrobial activity against *Escherichia coli D31*, carrying out its functions through active cell secretion during infection events. *HLP2 *shows antibacterial activity towards both gram-positive and gram negative agents, including *Pseudomonas aeruginosa *[79], and anti-parasitic activity against the ectoparasite *A. ocellatum*, as demonstrated by incubating gill cell lines with the spores of this microorganism [29]

*HLP2 *also binds the lipopolysaccharide (LPS) [80,81], protecting cells from an endotoxic shock mediator, the tumor necrosis factor [82], and other inflammatory mediators [83]. All these features, combined with increased transcript levels in our experiment, confirm that *HLP2 *has a central role in modulating the immune system response as a proper immune effector.

*HLP1 *shows specific antimicrobial activity towards different microorganisms, such as *Micrococcus luteus*, *Bacillus megaterium*, *E. coli*, and *Candida albicans *[84-86]. Cho and colleagues [87] have highlighted the role of *HLP1 *as immune modulator in catfish, where it is present in the epithelial cells in a nonacetylated form. They showed that, after skin injury, *HLP1 *is proteolysed by cathepsin D to increase the levels of antimicrobial peptide parasin I. Contrary to effects caused by acute stress, chronic stress decreases the transcriptional levels of *HLP1 *in catfish before any signs of illness can occur. The downregulation of AMPPs transcripts may dramatically increase the susceptibility of fish to infectious diseases [31]. In support of our data, it has been shown that, in rainbow trout, acute stress, such as confinement for a short time (minute to hours), rapidly increases the concentration of plasmatic proteins and stimulates the innate immune system, rather than suppressing it, in order to protect the fish from possible injuries [88].

*Dicentracin *gene showed an increase in the number of mRNA copies in skin and gills after the acute stress, compared to the same tissues of control animals. The recovery phase restored transcript levels to the control values only in the skin (Figure 1). The higher expression of *dicentracin *gene in gills and skin suggests that this AMPP represents a first and immediate line of defense in combating pathogens and stressors since these tissues constitute the first physiological barriers of the animal. Furthermore, in anticipation of a stress, which often occurs during many routine farm practices (hauling, grading, etc.) the AMPP levels in the population would be upregulated so that immune defenses are stronger after the stress is imposed. Under this scenario, the population remains resistant to pathogens commonly present in a latent state in the population. However, we cannot extensively discuss the potential role of the increase in *dicentracin *mRNA levels based on the results of the present study as we do not know whether such an mRNA profile is consistent with the functional protein levels. Therefore, without protein data we cannot draw any conclusions about the relationship between the mRNA levels and the *dicentracin *antimicrobial activity in gills and skin of stressed sea bass. Thus, our hypothesis that *dicentracin *has a role in the acute stress response in fish must be confirmed by further proteomic investigations.

## Conclusions

In conclusion, we isolated the sea bass cDNA sequences encoding for *Hb-LP*, *HLP1*, and *HLP2 *antimicrobial peptides in the present study. We have also shown that acute crowding stress induces an increase in the expression levels of the aforementioned genes and *dicentracin *in gills and skin of sea bass, and that this pattern of expression returned to basal levels within 24 hours of recovery in normal conditions. Future studies are necessary, however, to completely elucidate the underlying mechanism of AMPPs activation in marine teleosts. The present study is the first to investigate the role of these gene transcripts during acute confinement stress in a representative of this species.

## Authors' contributions

GT designed the study, participated in fish sampling, oversaw mRNA preparation, molecular cloning and sequencing, performed real time RT-PCR analysis, and phylogenetic analysis, and drafted the manuscript; AGC performed protein *in silico *analysis (protein annotation, tertiary structure, sites for post-translational modifications), and participated in the writing of the manuscript; EP participated in sampling, molecular cloning and sequencing, and mRNA extraction; GB and MS participated in planning the study. All authors read and approved the final manuscript.
